# Prevalence and risk factors for Human T-Lymphotropic Virus Type 1 (HTLV-1) among maintenance hemodialysis patients

**DOI:** 10.1186/s12882-017-0484-y

**Published:** 2017-02-15

**Authors:** Rilma F. S. Santos, Gildásio C. Conceição, Márcia S. Martins, Angiolina Kraychete, Maria A. C. Penalva, Edgar M. Carvalho, Antonio Alberto Lopes, Paulo Novis Rocha

**Affiliations:** 1 Division of Nephrology, Hospital Geral Roberto Santos, Salvador, Brazil; 2 Postgraduate Program in Health Sciences, Federal University of Bahia, Salvador, Brazil; 3 Department of Biochemistry, APAE, Salvador, Brazil; 4 Unit of Clinical Epidemiology and Evidence Based Medicine of the Professor Edgard Santos University Hospital, Federal University of Bahia, Salvador, Brazil; 5 Diretoria Médica da Clínica Nephron-Itapuã, Salvador, Brazil; 6 Centro de Pesquisas Gonçalo Moniz (Fiocruz-Ba), Salvador, Brazil; 7 Department of Internal Medicine and Diagnostic Support, Medical School of Bahia, Federal University of Bahia, Salvador, Brazil

**Keywords:** Blood Transfusion, Chronic Kidney 47 Disease, Hemodialysis, Hepatitis B, Hepatitis C, Human T-Lymphotropic Virus Type 1

## Abstract

**Background:**

Infection with the human T-cell lymphotropic virus type 1 (HTLV-1), although asymptomatic in most cases, can lead to potentially grave consequences, such as adult T-cell leukemia-lymphoma and HTLV-1-associated myelopathy / tropical spastic paraparesis. Its prevalence varies widely across different populations and geographic regions. A population-based study in the city of Salvador, located in the Northeast region of Brazil, showed an overall prevalence of HTLV-1 seropositivity of 1.7%. Blood borne virus infections are recognized as important hazards for patients and staff in maintenance hemodialysis (MHD) units but most studies focus on hepatitis B, hepatitis C and human immunodeficiency viruses. There are scarce data about HTLV-1 infection in the MHD population. We aimed to determine the prevalence and risk factors for HTLV-1 infection among MHD patients in the city of Salvador-Bahia, Brazil.

**Methods:**

We conducted a multi-center, cross-sectional study nested in a prospective cohort of MHD patients enrolled from four outpatient clinics. HTLV-1 screening was performed with ELISA and positive cases were confirmed by Western Blot. Factors associated with HTLV-1 seropositivity were identified by multivariable logistic regression.

**Results:**

605 patients were included in the study. The overall prevalence of HTLV-1 infection was 2.48% (15/605), which was similar to that of hepatitis B [1.98% (12/605)] and C [3.14% (19/605)] viruses in our sample. HTLV-1 seropositivity was positively associated with age [prevalence odds ratio (POR) 1.04; 95% confidence interval (CI) 1.01–1.08], unmarried status (POR 3.65; 95% CI 1.13–11.65), and history of blood transfusion (POR 3.35; 95% CI 1.01–11.13).

**Conclusions:**

The overall prevalence of HTLV-1 infection in a sample of MHD patients was similar to that of other viral infections, such as hepatitis B and C. Our data revealed that MHD patients who are older, unmarried or who have received blood transfusions are at higher risk for HTLV-1 infection.

## Background

The human T-cell lymphotropic virus type 1 (HTLV-1), the first human retrovirus identified [1], is the etiologic agent of adult T-cell leukemia-lymphoma (ATLL) and HTLV-1-associated myelopathy / tropical spastic paraparesis (HAM/TSP) [2–5]. HAM/TSP is the most severe form of neurologic manifestation of HTLV-1 infection, but a large percentage of infected subjects present with milder forms of neurologic involvement, such as erectile dysfunction or neurogenic bladder [6, 7]. Patients with HTLV-1-related neurogenic bladder may present with urinary symptoms that mimic urinary tract infections [8] and ultimately evolve to obstructive kidney failure [9]. More recently, HTLV-1 infection has been linked to other manifestations, such as uveitis, Sicca syndrome, chronic periodontitis, infective dermatitis, polymyositis and polyneuropathy [10–12], although most seropositive patients remain asymptomatic for prolonged periods.

HTLV-1 infection has become a worldwide epidemic, but studies focusing on population prevalence are scarce and stem mainly from endemic areas [13]. The distribution of the virus is not homogeneous and its prevalence varies according to the geographic area and characteristics of the population, with a spatial variation within each region [13–15]. Data obtained mainly from non-population-based studies indicate that the areas with the highest prevalence of HTLV-1 infection are Southern Japan, Caribbean islands, west and central regions of Africa, Malanesia islands, some regions of the Middle East and some regions of South America [16–19].

In Brazil, most of the prevalence data have been obtained from serological screening of asymptomatic blood donors. These data show a great variation in prevalence within the country, being highest in the northeast region [20–23]. In northeast Brazil, the highest prevalence of HTLV-1 infection is observed in the city of Salvador, capital of the state of Bahia. In a population-based study, the overall prevalence of HTLV-1 seropositivity in Salvador was 1.7% [95% CI 1.1–2.5%] [24].

Blood borne virus infections are recognized as important hazards for patients and staff in maintenance hemodialysis (MHD) units but most studies focus on hepatitis B (HBV), hepatitis C (HCV) and human immunodeficiency virus (HIV). Few studies were specifically designed to estimate the prevalence of HTLV-1 infection in MHD patients [25–30]. These studies, conducted in the last century, revealed that, similarly to what is observed in the general population, the prevalence of HTLV-1 infection among MHD patients varies according to the geographic area. In a study of patients undergoing MHD in several regions of Japan, the average prevalence of HTLV-1 infection was 2.7% but there was a wide variation across regions, reaching up to 21.2% in Okinawa, a highly endemic area of HLTV-1 among asymptomatic blood donors [26]. A more recent study, conducted in the Mazandaran province in north Iran, a non-endemic region of HLTV-1, found a prevalence of HTLV-1 of 0.6% [31]. We found no published data about the prevalence of HTLV-1 in MHD patients treated in Brazil.

Given the high prevalence of HTLV-1 infection in the general population in Salvador-Bahia and the scarcity of data about this infection in the MHD population, we conducted a cross-sectional study nested in a prospective, multicenter cohort of MHD patients, to identify the prevalence and factors associated with HTLV-1 seropositivity in this population.

## Methods

### Subjects and sites

We screened MHD patients enrolled in the Prospective Study of the Prognosis of Chronic Hemodialysis Patients (PROHEMO) cohort. The current phase of the PROHEMO study cohort began in 2010; it includes MHD patients from four (out of a total of twelve) outpatient dialysis units in the city of Salvador, the capital of Bahia, located in the northeast region of Brazil [32, 33]. We do not have an epidemiologic profile of the remaining eight clinics, but we believe the four clinics studied to be representative of all clinics in Salvador. We included all adult (18 or older) members of the PROHEMO cohort who were alive and undergoing MHD at the time of the collection of blood samples for HTLV-1 serology screening (between August and October 2011). At this time, the cohort contained 695 patients; we excluded 90 patients due to lack of essential clinical data. Therefore, our analyses were conducted on 605 patients, corresponding to 87% (605/695) of the entire cohort.

### Design

Cross-sectional study nested in a prospective cohort study of MHD patients. Information on socio-demographic, laboratorial and clinical variables ware obtained directly from the PROHEMO database, which contains data prospectively collected from patients and medical records. Information on HTLV-1 status was obtained by screening stored blood samples. Between August and October 2011, at the time of blood collection for monthly dialysis labs, additional 10 ml samples of blood were drawn and stored at −20 °C for future analyses. These samples were screened for antibodies against HTLV 1/2, using an enzyme immunoassay (EIA) - Gold ELISA HTLV-1/2 (REM Indústria e Comércio LTDA) and the positive cases were confirmed by a Western Blot (WB) immunoassay (HTLV Blot 2.4, MP Biomedicals Asia Pacific Pte Ltd. Singapore).

The economic class of the patients (A, B, C, D and E) was determined using the classification system of the Brazilian Institute of Market Research (Abipeme) that considers consumer goods, such as refrigerators, televisions and telephones. Patients in classes D and E were categorized as poor or very poor [34].

A variable “history of blood transfusion” was available in the dataset. Participants of the cohort were asked the following question “Did you receive blood transfusion over the past three months?” There were no data on transfusions past this period or on the number of units transfused.

### Ethical issues

All participants signed an informed consent form agreeing with the use of their clinical information and collection and testing of blood samples for HTVL serology for research purposes. The medical director of each participating dialysis unit signed a consent letter approving the study protocol. The Institutional Review Board of the Medical School of Bahia of the Federal University of Bahia approved the study protocol.

### Sample size calculation

In December 2011, the population of MHD patients of the city of Salvador was estimated to be around 2000 patients (unpublished data from the State’s Health Secretariat). Using the software Openepi (www.openepi.com), we estimated that 548 patients would be needed to detect a prevalence of HTLV-1 seropositivity in MHD patients in Salvador of 2%, with 95% confidence limits extending 1% on either side.

### Statistical analyses

Continuous data were summarized using mean ± standard deviation (SD) or median and interquartile range (IQR); categorical variables were summarized using relative frequencies. Comparisons of continuous variables between seropositive and seronegative groups were performed using the Student’s *t*-test for independent samples; categorical variables were compared using the chi square (with continuity correction) or Fischer’s exact test. Variables that showed an association with HTLV-1 seropositivity characterized by a *p* value ≤ 0.25 in univariate analyses were selected for a multivariable logistic regression model to identify independent predictors of HTLV-1 seropositivity. We used an automated multivariable logistic regression method called forward stepwise. Briefly, in this method the computer tests the variables one by one for entry into the model, based on the significance level of the score statistic. The variable with the smallest *p* value is entered into the model (entry criterion: *p* < 0.05). After each entry, variables that are already in the model are tested for possible removal. The variable with the largest *p* value is removed (removal criterion: *p* > 0.10), and the model is re-estimated. Variables in the model are then evaluated again for removal. When no more variables satisfy the removal criterion, covariates that are not in the model are evaluated for entry. Model building stops when no more variables meet entry or removal criteria or when the current model is the same as a previous model. A *p* value ≤ 0.05 in final analyses was considered statistically significant. The analyses were performed using SPSS for Windows version 21.0 and the 95% confidence interval of the HTLV-1 prevalence was calculated using the bootstrap (50 replications) of the STATA, version 12.0.

## Results

Sixteen patients were HTLV-1 seropositive by the ELISA technique but one was considered a false positive due to a negative Western Blot. Therefore, 15 out of 605 MHD patients studied had confirmed HTLV-1 infection, for an overall prevalence of 2.48% (95% CI 1.22–3.74%). There were no cases of HTLV-2 seropositivity in our sample.

In two out of these fifteen cases (13.3%), the ESRD was attributed to urological complications of HTLV-1 infection, specifically neurogenic bladder leading to obstructive kidney failure. The first case was a 70 year-old female, wheelchair-bound due to HAM/TSP secondary to HTLV-1. She had a well-documented case of neurogenic bladder and was followed by a team of Neurologists and Urologists at an ambulatory clinic specialized in HTLV-1 at the University Hospital of the Federal University of Bahia. She did not have diabetes or hypertension and other causes of ESRD were excluded on clinical grounds. The second case was a 47 year-old man who initially presented to the emergency department of a public general hospital with uremia. He was found to have a distended bladder and bilateral hydronephrosis on ultrasound, with a normal-sized prostate gland. A Foley catheter was placed, but renal failure did not improve and he remained dialysis-dependent. He had no prior history of hypertension or diabetes.

Table 1 shows the socio-demographic, clinical and laboratorial characteristics of our sample, stratified by HTLV-1 serology. In this univariate analysis, unmarried status and a history of blood transfusion were the only variables significantly associated with a positive HTLV-1 serology. The mean age tended to be greater and time on dialysis tended to be lower in the seropositive group. Overall, there were 12/604 patients (1.99%) with HBV and 19/605 (3.14%) with HCV; the frequency of positive HBV or HCV serology tended to be higher in the HTLV-1 group. Seropositive patients also had higher serum creatinine, higher parathyroid hormone (PTH) and lower leucocyte count whereas hemoglobin levels did not differ among groups (data not shown).

**Table 1:** Demographic, clinical and laboratorial variables of 605 maintenance hemodialysis patients from four outpatient dialysis units in Salvador, Bahia, Brazil, stratified by HTLV-1 serology

Independent variables	HTLV (+) *N* = 15	HTLV (−) *N* = 590	POR (95% CI)	*p*
Demographic				
Age, years	52.5 (46.9–71.0)	47.8 (37.7–59.0)	1.03 (1.00–1.07)	**0.080**
Female (vs. male)	8/15 (53.3)	224/590 (38.0)	1.87 (0.67–5.22)	**0.234**
Non-white (vs. white)	13/15 (86.7)	513/590 (86.9)	1.03 (0.23–4.63)	0.974
Poor or very poor (vs. classes A thru C)	10/15 (66.7)	265/580 (45.7)	2.38 (0.80–7.04)	**0.118**
Less than high school (vs. high school or greater)	12/15 (80.0)	359/589 (61.0)	2.56 (0.72–9.18)	**0.148**
Unmarried (vs. married)	11/15 (73.3)	261/588 (44.4)	3.45 (1.09–10.95)	**0.036**
Clinical				
Dialysis by catheter (vs. AV fistula)	4/15 (26.7)	76/588 (12.9)	1.12 (0.31–4.02)	0.867
Time on MHD, months	20.4 (4.5–58.4)	47.8 (19.6–97.4)	0.99 (0.97–1.00)	**0.064**
EPO, IU (x10^3^)/week	12 (6–12)	10 (6–12)	1.00 (1.00–1.00)	0.870
Diabetes mellitus (vs. no)	5/15 (33.3)	100/590 (16.9)	2.45 (0.82–7.32)	**0.109**
Systemic hypertension (vs. no)	13/15 (86.7)	533/590 (90.3)	0.70 (0.15–3.16)	0.638
Blood transfusion (vs. no)	4/15 (26.7)	55/582 (9.2)	3.48 (1.07–11.31)	**0.038**
Hepatitis B (vs. no)	1/15 (6.7)	11/589 (1.9)	3.75 (0.45–31.10)	0.220
Hepatitis C (vs. no)	1/15 (6.7)	18/590 (3.1)	2.27 (0.28–18.21)	0.440
Hepatitis B or C (vs. no)	2/15 (13.3)	28/589 (4.8)	3.08 (0.66–14.33)	**0.151**

Continuous data (Age, Time on MHD and EPO dose) are expressed as median (P25–P75%); categorical data are expressed as n/total (%); *POR* Prevalence Odds Ratio, *EPO* Erythropoietin, *MHD* maintenance hemodialysis. For continuous data, POR reflects the odds of a positive HTLV serology with each increase in one unit of the continuous variable (one year for Age, one month for Time on MHD, and 10^3^ IU/week for EPO); for categorical data, POR reflects the odds of a positive HTLV serology in the variable of interest compared to its complement. History of blood transfusion was limited to available data that reported transfusion only for the previous three months. *P* values in bold highlight the variables that were selected for the multivariable logistic regression model. Although a positive serology for Hepatitis B was associated with a *p* ≤ 0.25, it was not included in the multivariable model because we preferred to use the variable Hepatitis B or C

**Fig. 1:**
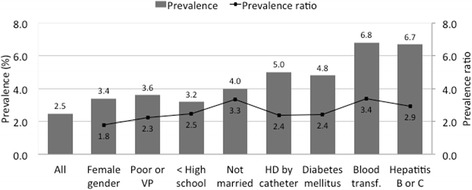
Prevalence and prevalence ratio of HTLV-1 seropositivity in subgroups of maintenance hemodialysis patients from four satellite clinics in Salvador, Bahia, Brazil

Figure 1 shows the prevalence and prevalence ratio of HTLV-1 seropositivity in subgroups of MHD patients. The highest prevalence ratios were encountered in those who received blood transfusions, unmarried and with HBV or HCV. The prevalence of HTLV-1 infection also increased with age, going from 1.6 to 2.2% and 2.9% among those 18–30, 31–50 and > 50 years old, respectively.

Table 2 shows the multivariable logistic regression to identify variables independently associated with HTLV-1 seropositivity. In this analysis, the variables that showed statistically significant associations with HTLV-1 seropositivity were older age, unmarried status and a history of blood transfusion.

**Table 2:** Multivariable logistic regression to identify variables independently associated with HTLV-1 seropositivity in 605 maintenance hemodialysis patients from four satellite clinics in Salvador, Bahia, Brazil

Independent variables	POR	95% CI		*p*
		Lower	Upper	
Age, years	1.04	1.01	1.08	0.041
Unmarried (vs. married)	3.65	1.13	11.75	0.030
Blood transfusion (vs. no)	3.35	1.01	11.13	0.048

Nine variables were selected to enter the automated, forward stepwise multivariable logistic regression model based on a *p* value ≤ 0.25 on univariate analyses: age (continuous), gender (female as reference), marital status (unmarried as reference), time on dialysis, history of blood transfusion (yes/no), seropositivity for hepatitis B or C virus (yes/no), socioeconomic status (“poor or very poor” as reference), level of schooling (“less than high school” as reference), and diabetes (yes / no). History of blood transfusion was limited to available data that reported transfusion only for the previous three months

## Discussion

Our initial hypothesis was that the prevalence of HTLV-1 in the MHD population would be greater than that observed in the general population. This hypothesis was based on older literature data from other countries [27–29, 35] and on the following postulates: a) ESRD could be a risk factor for HTLV-1, mediated by anemia and greater frequency of blood transfusion or due to the risk of acquiring the virus during the hemodialysis procedure, as it is known to happen with HBV and HCV [36, 37]; b) HTLV-1 and end stage renal disease (ESRD) could share risk factors, such as poverty and health illiteracy [38, 39]; c) HTLV-1 could be a risk factor for ESRD as the urological manifestations of the virus, such as neurogenic bladder, can lead to post-renal kidney failure [9]. Nevertheless, the overall prevalence of HTLV-1 seropositivity in our sample of 605 MHD patients was 2.48% (95% CI 1.22–3.74%), which is only marginally higher than that observed by Dourado et al. in a population-based study in the city of Salvador (1.7; 95% CI 1.1–2.5%) [24].

In their study, Dourado et al. studied individuals from 1 to 89 years. The prevalence of HTLV-1 infection increased with age, going from 0.3 to 1.1 to 1.1 and 8.4% among those 0–15, 16–30, 31–50 and > 50 years old, respectively. In the present study of MHD patients, the prevalence of HTLV-1 infection also increased with age, going from 1.6% in those 18–30 to 2.9% among those > 50 years old; for every increase in one year of age, we found a 4% increase in the odds of having a positive HTLV-1 serology. A potential explanation for this association is that increasing age provides a greater length of exposure to events that might result in acquiring the virus, such as sexual activity, intravenous drug use or blood transfusion. Since there is no cure for HTLV-1, once acquired, the infection is carried forward over time (cohort effect).

The association between HTLV-1 infection and blood transfusion is well established and has been confirmed in several studies using blood donors in different countries [39–41], including Brazil [38]. Blood transfusions among MHD patients were much more common before the advent of recombinant human erythropoietin (rhEPO) [42, 43]; indeed, this might help explain the high prevalence of HTLV-1 in the MHD population of some Japanese studies published before the advent of rhEPO [25, 26]. Currently, the routine use of rhEPO markedly reduced the need for blood transfusion, particularly among those patients who are already established on MHD programs for more than three months [44].

In Brazil, however, there is still a considerable need for blood transfusions among patients who are recent on MHD, since many of them start dialysis emergently, without the benefit of pre-dialysis medical care. This was illustrated in a study conducted at the largest public hospital in Salvador [45], which is a referral service for the entire state of Bahia. The authors studied all patients who initiated MHD at that institution after being admitted through the emergency department and found that 57% of them first learned about their kidney disease at the time of diagnosis of ESRD. Moreover, 71% of them had not been evaluated by a nephrologist before arriving at the emergency department. Their mean hemoglobin at admission was 7.7 mg/dL; no patient had received rhEPO previously and 50% of them required blood transfusion during the hospitalization [45]. In the present study, we found that a history of blood transfusion tripled the odds of having a positive HTLV-1 serology. Spread of HTLV-1 during the hemodialysis procedure seems less likely, based on the biology of the virus. Some studies indicate that transmission of HTLV-1 requires the contact between living cells and not simply cell-free body fluids [46, 47].

In their population-based study, Dourado et. al showed that the prevalence of HTLV-1 was greater among females, those with lower income, worse living conditions and less education [24]. In the present study of the MHD population, we also found a numerically higher prevalence of HTLV-1 infection among females, those who were poor or very poor and those who had less than high school education, although these associations did not reach statistical significance. Unmarried status, however, more than tripled the odds of having a positive HTLV-1 serology. We posit that unmarried status might have functioned as a proxy for sexual promiscuity and/or unprotected sex, which are known risk factors for HTLV-1 infection [38, 39].

In Brazil, the main etiologies of ESRD are hypertension (35%), diabetes mellitus (30%), chronic glomerulonephritis (12%) and polycystic kidney disease (4%) [48]. Herein, urological complications of HTLV-1 infection were felt to be the cause of ESRD in only 0.3% of patients (2/605). We are not aware of other studies that evaluated the contribution of HTLV-1 to ESRD.

Our study has several limitations. An inherent limitation of cross sectional studies is that associations do not imply causality. We utilized the database of an ongoing multicenter cohort study of MHD in our area and all of our analyses were limited to the variables that were present in their database. We did not have information on sexual practices, living conditions, intravenous drug use or neurologic symptoms. Moreover, our data on blood transfusion was categorical (yes/no) and restricted to the previous three months; we had no information on lifetime exposure to blood products. Finally, our study provides no information about HIV co-infection as none of the four MHD units studied admit HIV positive patients.

## Conclusion

The prevalence of HTLV-1 infection in MHD patients from four hemodialysis units in northeast Brazil was not higher than that encountered in a population-based study of the same geographic area. HTLV-1 seropositivity in MHD patients was similar to that of other viral infections, such as HBV and HCV. Older age, unmarried status and blood transfusions were independently associated with HTLV-1 infection.

## Abbreviations

ATLL: Adult T-cell leukemia-lymphoma
Blood Transfusion: Chronic Kidney Disease, Hemodialysis, Hepatitis B, Hepatitis C, Human T-Lymphotropic Virus Type 1
ESRD: End stage renal disease
HAM / TSP: HTLV-1-Associated Myelopathy / Tropical Spastic Paraparesis
HBV: Hepatitis B virus
HCV: Hepatitis C virus
HIV: Human immunodeficiency virus
HTLV-1: Human T-lymphotropic virus type 1
MHC: Maintenance hemodialysis
PROHEMO: Prospective study of the prognosis of chronic hemodialysis patients
PTH: Parathyroid hormone
RhEPO: Recombinant human erythropoietin
